# *Borrelia miyamotoi* Infections in Small Mammals, California, USA

**DOI:** 10.3201/eid2412.171632

**Published:** 2018-12

**Authors:** Daniel J. Salkeld, Nathan C. Nieto, Denise L. Bonilla, Melissa H. Yoshimizu, Kerry A. Padgett

**Affiliations:** Colorado State University, Fort Collins, Colorado, USA (D.J. Salkeld);; Northern Arizona University, Flagstaff, Arizona, USA (N.C. Nieto);; California Department of Public Health, Richmond, California, USA (D.L. Bonilla, M.H. Yoshimizu, K.A. Padgett)

**Keywords:** *Borrelia miyamotoi* ecology, tick-borne disease, mammals, *Peromyscus*, California, vector-borne infections, bacteria, Borrelia, United States, zoonoses

## Abstract

Surveillance to investigate the wildlife–vector transmission cycle of the human pathogen *Borrelia miyamotoi* in California, USA, revealed infections in dusky-footed woodrats, brush mice, and California mice. Phylogenetic analyses suggest a single, well-supported clade of *B. miyamotoi* is circulating in California.

*Borrelia miyamotoi* is a spirochete that causes a relapsing febrile illness and is transmitted by hard *Ixodes* species ticks (*1*,*2*). *B. miyamotoi* is prevalent in western black-legged tick (*I. pacificus*) populations in California, USA (*3**,**4*); in some locations, *B. miyamotoi* prevalence in ticks is comparable with or higher than the prevalence of the Lyme disease agent, *B. burgdorferi* sensu stricto (*3*–*5*). There is mounting evidence that human infections occur in northern California (*6*).

Surveillance of *B. miyamotoi* in California has focused on ticks, and little is known about infection in wildlife hosts. *B. miyamotoi* has been identified from spleen samples of birds and rodents in Europe (*7*), from blood and bladder samples of rodents in Japan (*8*), and from white-footed mice (*Peromyscus leucopus*) in the eastern United States (*9*). In California, surveillance in Alameda County (east of the San Francisco Bay) observed *B. miyamotoi* in tick populations but failed to detect the spirochete in mammals (*10*). We investigated *B. miyamotoi* infection status in small mammals at 3 California sites where the bacterium is present in tick populations (*3**,**5*).

## The Study

We captured animals on 2 consecutive nights at each of 3 sites in the San Francisco Bay area of California during June 2014: Windy Hill (37.37315°, −122.22466°) and Thornewood (37.39086°, −122.25066°) Open Space Preserves (OSP) in San Mateo County, and Foothills Park (37.36243°, −122.17362°) in Santa Clara County. We chose these sites on the basis of local *B. miyamotoi* prevalence of 3.6%–10.7% in *I. pacificus* nymphs (*5*). Trapping occurred in June to coincide with the peak abundance of nymphal black-legged ticks and so perhaps increase the chance that *B. miyamotoi* would be circulating in animal populations (*11*). Animals were captured using Sherman live traps baited with peanut butter and oats. 

We anesthetized captured animals with isoflurane, identified them by morphology using taxonomic guides, and examined them for ticks. We obtained blood and ear-tissue biopsy samples from each individual and tested both sample types for *Borrelia* spp. because different *Borrelia* species may vary in tissue tropism (*9*).

We extracted DNA from all samples (i.e., whole blood, ear punch biopsies, and ticks) using DNeasy Blood and Tissue Kits (QIAGEN, Valencia, CA, USA) and assayed for the presence of *Borrelia* using quantitative PCR (qPCR) (*9*), which is able to detect as few as 10 spirochetes. We sequenced all qPCR-positive samples using a primer set targeting the intergenic spacer *rrs-rrlA* locus, which allowed for differentiation of *Borrelia* genospecies (*12*). Alignments were made in ClustalX (http://www.clustal.org). We compared our sequences from *I. pacificus* ticks and wild-caught rodents (GenBank accession nos. MH342008–31) to representative GenBank sequences from isolates found in other sites in California (accession nos. KT343321, KT343334, KT343337, KU184505, KF957668), elsewhere in the United States (accession nos. HQ658901, HQ658902, AY374140, AY37139, AY374138, AY363706, GU993308, KY293400, KY293399, KY293398, KY293397, KY293396, GQ856588, GU993309, GQ856589), Japan (accession nos. AY363703, AY363704), and Sweden (accession no. AY363705). We conducted phylogenetic reconstruction using MrBayes (http://mrbayes.csit.fsu.edu/) and visualizations using FigTree (http:// tree.bio.ed.ac.uk/software/figtree/).

We captured a total of 117 small mammals from 5 species (Table 1). Our surveillance efforts demonstrate that *B. miyamotoi* infects woodrats (*Neotoma fuscipes*), brush mice (*Peromyscus boylii*), and California mice (*P. californicus*) (Table 2). At sites where *B. miyamotoi* was present in small mammals, *B. miyamotoi* prevalence was 6%–33% in different host species (Table 2). These data reflect *B. miyamotoi* prevalence in small-mammal hosts in other geographic regions: 10.7% of voles and mice (n = 65) in the Netherlands, where nymphal infection prevalence (NIP) of *B. miyamotoi* in *I. ricinus* ticks is 2.5% (84/3360) (*7*), and 6.5% of white-footed mice (*P. leucopus*) in the northeastern United States, where NIP in *I. scapularis* ticks is 5.5% (38/689) (*9*). We did not observe *B. miyamotoi* in pinyon mice or deer mice, either because of small sample sizes or because these species are not involved in *B. miyamotoi* transmission. In nearby Alameda County, *B. miyamotoi* was not observed in small mammals (*10**)*; possible reasons are that the spirochete is rarer in this locality (NIP = 0.4% in Alameda study sites), that mammal capture periods were dispersed across multiple years and not as coincident with nymphal tick activity, or that brush mice and California mice were not captured at that location.

**Table 1:** Numbers of mammals captured and tested for *Borrelia* spp., by species and location, California, USA*

Species	Foothills Park	Thornewood OSP		Windy Hill OSP	Total
		Redwood habitat	Oak–madrone woodland		
Dusky-footed woodrat (*Neotoma fuscipes*)	4	0	1	1	6
Brush mouse (*Peromyscus boylii*)	27	9	17	18	71
California mouse (*Peromyscus californicus*)	6	3	9	6	24
Deer mouse (*Peromyscus maniculatus*)	6	4	2	1	13
Pinyon mouse (*Peromyscus truei*)	3	0	0	0	3
Total	46	16	29	26	117

*OSP, Open Space Preserves.

**Table 2:** Prevalence of *Borrelia spp.* in small mammal species in the San Francisco Bay area, California, USA

*Borrelia* species	Mammal species	Site	Sample source	Prevalence at site, no. tested/no. positive (%)	Prevalence across sites, no. tested/no. positive (%)
*B. miyamotoi*	Dusky-footed woodrat	Foothills	Blood	1/4 (25)	1/6 (17)
	Brush mouse	Thornewood woodland	Ear	1/17 (6)	2/71 (3)
	Brush mouse*	Windy Hill	Ear	1/18 (6)	
	California mouse	Foothills	Ear	1/6 (17)	4/24 (17)
	California mouse	Thornewood woodland	2 ear, 1 blood	3/9 (33)	
*B. bissettiae*	Brush mouse	Thornewood woodland	Ear	1/17 (6)	1/71 (1)
	Pinyon mouse	Foothills	Blood	1/3 (33)	1/3 (33)
*B. burgdorfer*i sensu lato	California mouse	Windy Hill	Blood	1/6 (17)	1/24 (4)
*Borrelia spp.*, not sequenced	Brush mouse	Thornewood woodland	2 blood, 1 blood and ear	3/17 (18)	3/71 (4)

*Co-infection with *B. burgdorferi* sensu lato.

Other identified *Borrelia* species included *B. bissettiae* in 1 pinyon mouse (*P. truei*) and 1 brush mouse, which mirrors earlier studies of *B. bissettiae* from farther north in California (*13**,**14*). Woodrats, California voles (*Microtus californicus*), deer mice (*P. maniculatus*), and black rats (*Rattus rattus*) have also been observed infected with *B. bissettiae* (*10**,**13*). During previous tick sampling efforts at our study sites, we did not detect *B. bissettiae* in questing western black-legged ticks (*3**,**5*).

We did not find *B. burgdorferi* sensu stricto, although we observed uncharacterized *B. burgdorferi* sensu lato infection in 1 California mouse. None of the animals captured in the redwood habitat (Thornewood OSP) were infected with *Borrelia* spp., although the sample size was small at this location (Table 1). We found a co-infection of *B. burgdorferi* sl and *B. miyamotoi* in a brush mouse. Co-infections of *B. burgdorferi* and *B. miyamotoi* have previously been reported from mice and ticks in the northeastern United States (*9*) and from ticks in Marin County, California (*4*).

Five mammals were infested with *I. angustus* ticks, of which we observed all 3 life stages. In redwood habitat at Thornewood OSP, 1 California mouse hosted 2 adult females and a nymph, 1 brush mouse was infested with 2 adult females and 2 nymphs, and 1 brush mouse carried 1 female and 1 nymphal tick. Of 2 brush mice captured at Windy Hill, 1 harbored 3 larvae and the other 5 larvae. None of the 17 *I. angustus* ticks tested positive for *Borrelia* spp., nor did the host animals from which the ticks were removed. We found no *I. pacificus* ticks infesting the small mammals.

Phylogenetic analyses suggest that *B. miyamotoi* in California is a single strain, separate from *B. miyamotoi* in the eastern United States and from strains circulating in Asia and Europe (Figure), corroborating an earlier study (*15*). Sequences from *I. pacificus* ticks previously collected in the San Francisco Bay area were identical to the sequences obtained from rodent infections.

**Figure:**
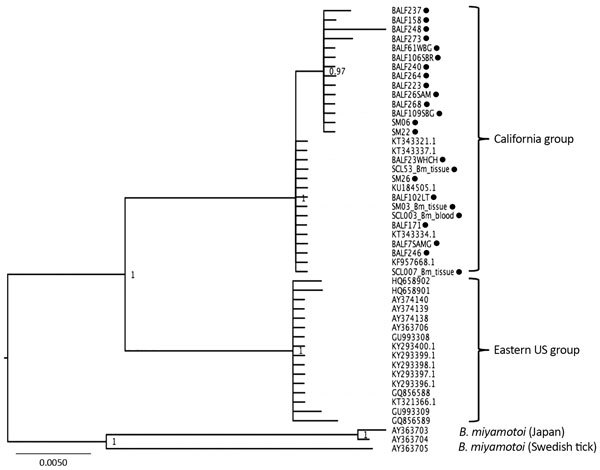
Phylogenetic tree of *Borrelia miyamotoi* intergenic spacer (*rrs-rrlA*) sequences isolated from wild-caught rodents and ticks (black dots) from California, USA, in study of *Borrelia spp.* in small mammal species in the San Francisco Bay area, compared with reference samples from California, the eastern United States, Japan, and Sweden. Isolates are identified by isolate identification number or GenBank accession number. Scale bar indicates nucleotide substitutions per site.

## Conclusions

The identification of *B. miyamotoi* in small mammals in California mirrors research from other locations that have documented the spirochete in small rodents. It is premature to claim these infected species as *B. miyamotoi* reservoirs (i.e., responsible for maintenance of the pathogen and acting as a source for of zoonotic transmission), in part because *B. miyamotoi* can also be transmitted transovarially in the tick population; local maintenance of the spirochete may not require a reservoir host. Nonetheless, in California, where *B. miyamotoi* clusters into a single, well-supported phylogenetic clade, the spirochete appears to be circulating among rodent species. Xenodiagnostic investigation of these putative reservoir species, as well as more comprehensive investigations of the reservoir potential of other local fauna, including larger mammals (e.g., squirrels and deer) and birds, will further elucidate the ecology of *B. miyamotoi* in California.
